# *cis*-Dichlorido[4,4,5,5-tetra­methyl-2-(2-pyrid­yl)-2-imidazoline-1-ox­yl]­palladium(II) tetra­hydro­furan hemi­solvate

**DOI:** 10.1107/S1600536808004406

**Published:** 2008-02-20

**Authors:** Sihem Badeche, Djamil Azzedine Rouag, Sabrina Benmebarek, Salah-Eddine Bouaoud, Stéphane Golhen

**Affiliations:** a Laboratoire de Chimie Moléculaire, du Contrôle de l’Environnement et de Mesures Physico-Chimiques, Département de Chimie, Université Frères Mentouri, Constantine, Algeria; b Sciences Chimiques de Rennes, UMR 6226, CNRS–Université de Rennes 1, 263 Avenue du Général Leclerc, CS 74205, 35042 Rennes Cedex, France

## Abstract

The asymmetric unit of the title complex, [PdCl_2_(C_12_H_16_N_3_O)]·0.5C_4_H_8_O, consists of one palladium complex in a general position and one half tetra­hydro­furan (THF) solvent mol­ecule, with the O atom lying on a twofold rotation axis. The Pd^II^ atom is bound to one chelating imino nitroxide radical through two N atoms, one from the pyridyl ring and the other from the imidazoline ring. The coordination of the metal centre is completed by two Cl atoms in a *cis* configuration, leading to a quasi-square-planar coordination of the metal centre. The four atoms that define the Pd^II^ coordination environment and the eight atoms that belong to the pyridylimine fragment are coplanar, with no deviation larger than 0.087 (5) Å. In the crystal structure, inter­molecular inter­actions shorter than the corresponding van der Waals radii sum are observed only between Pd^II^ complexes, and no short contact is observed around the THF mol­ecule. Weak C—H⋯O and C—H⋯Cl inter­actions yield a two-dimensional network of complexes in the (101) plane.

## Related literature

For related literature, see: Caneschi *et al.* (1991 ▶); Davis *et al.* (1972 ▶); Evans *et al.* (1968 ▶); Fettouhi *et al.* (2003 ▶); Li *et al.* (2004 ▶); Ma *et al.* (2006 ▶, 2007 ▶); Oshio *et al.* (1996 ▶); Ueda *et al.* (2003 ▶, 2005 ▶); Ullman & Holm (1970 ▶); Xu *et al.* (2007 ▶).
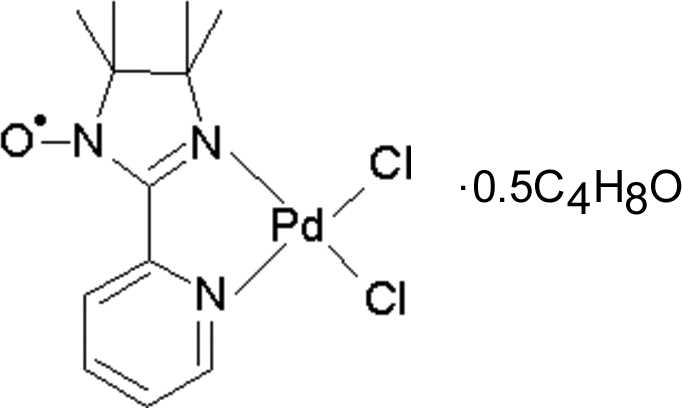


## Experimental

### 

#### Crystal data

[PdCl_2_(C_12_H_16_N_3_O)]·0.5C_4_H_8_O
*M* *_r_* = 431.65
Monoclinic, 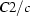

*a* = 19.1398 (10) Å
*b* = 15.2061 (12) Å
*c* = 13.8291 (10) Å
β = 123.415 (3)°
*V* = 3359.6 (4) Å^3^
*Z* = 8
Mo *K*α radiation
μ = 1.43 mm^−1^
*T* = 293 (2) K
0.7 × 0.3 × 0.3 mm

#### Data collection

Nonius KappaCCD diffractometer
Absorption correction: none
5707 measured reflections
3065 independent reflections
2049 reflections with *I* > 2σ(*I*)
*R*_int_ = 0.059

#### Refinement

*R*[*F* ^2^ > 2σ(*F* ^2^)] = 0.057
*wR*(*F* ^2^) = 0.152
*S* = 1.03
3065 reflections
199 parameters
H-atom parameters constrained
Δρ_max_ = 0.61 e Å^−3^
Δρ_min_ = −0.95 e Å^−3^

### 

Data collection: *COLLECT* (Nonius, 2000 ▶); cell refinement: *HKL* *SCALEPACK* (Otwinowski & Minor, 1997 ▶); data reduction: *HKL* *DENZO* (Otwinowski & Minor, 1997 ▶) and *SCALEPACK*; program(s) used to solve structure: *SHELXS97* (Sheldrick, 2008 ▶); program(s) used to refine structure: *SHELXL97* (Sheldrick, 2008 ▶); molecular graphics: *ORTEP-3 for Windows* (Farrugia, 1997 ▶) and *Mercury* (Macrae *et al.*, 2006 ▶); software used to prepare material for publication: *WinGX* (Farrugia, 1999 ▶).

## Supplementary Material

Crystal structure: contains datablocks global, I. DOI: 10.1107/S1600536808004406/dn2318sup1.cif

Structure factors: contains datablocks I. DOI: 10.1107/S1600536808004406/dn2318Isup2.hkl

Additional supplementary materials:  crystallographic information; 3D view; checkCIF report

## Figures and Tables

**Table 1:** Hydrogen-bond geometry (Å, °)

*D*—H⋯*A*	*D*—H	H⋯*A*	*D*⋯*A*	*D*—H⋯*A*
C4—H4⋯O1^i^	0.93	2.61	3.307 (8)	132
C10—H10*B*⋯Cl1^ii^	0.96	2.77	3.691 (7)	160

Symmetry codes: (i) 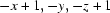; (ii) 
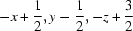
.

**Fig. 1.**
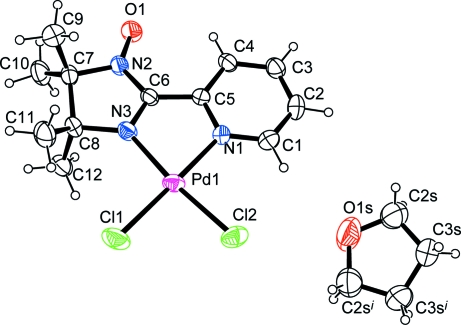
An ORTEP drawing of title compound showing 30% probability displacement ellipsoids and the atom-numbering scheme. The labels of the H atoms have been omitted for clarity. [Symmetry codes: (i) -x + 1, y, -z + 1.5]

**Fig. 2.**
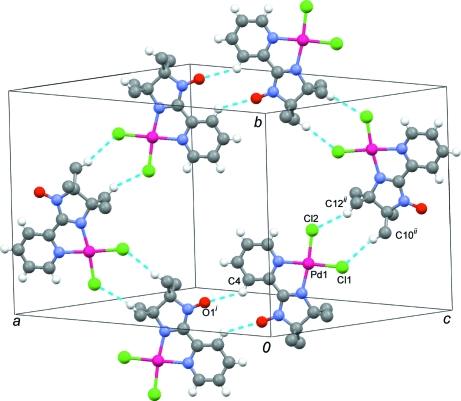
Partial packing view showing the formation of a two-dimensionnal network through C—H···O and C—H···Cl intermolecular interactions. H bonds are shown as dashed lines. H atoms not involved in hydrogen bonding have been omitted for clarity. [Symmetry codes: (i) -x –+1, -y, -z + 1; (ii) 1/2 - x, y + 1/2, 3/2 - z]

**Fig. 3.**
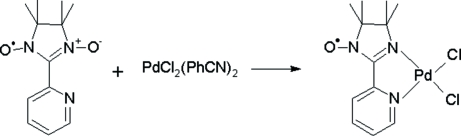
The formation of the title compound.

## supplementary crystallographic information

### Comment

Organic nitronyl nitroxide radicals have attracted much attention for their magnetic properties in general and ferromagnetism in particular. Then several transition metal complexes with stable nitronyl nitroxide radical ligands have been prepared and extensively investigated (Ma *et al.*, 2007; Xu *et al.*, 2007; Ma *et al.*, 2006; Ueda *et al.*, 2005; Li *et al.*, 2004; Ueda *et al.*, 2003; Oshio *et al.*, 1996; Caneschi *et al.*, 1991). Our contribution in this field is the synthesis (see Scheme 1) and structure characterization of the title compound.

The molecular structure of the complex [Pd(I*M*_2_py)Cl_2_] 0.5THF(I) is shown in Fig. 1 while selected geometric parameters are given in Table 1. Focusing the coordination square of Pd^II^, one can notice that Cl2 atom deviates significantly from the phane defined with atoms N3/N1/Pd1/Cl1 by 0.0843 (23) Å. The mean bond distance of Pd—Cl 2.277 Å is in agreement with the values observed in a similar complex while the average bond length of Pd—N 2.045 Å is slightly longer than seen previously (Fettouhi *et al.*, 2003). Due to a chelation of the iminonitroxide radical with Pd^II^ ion, the four atoms which define the Pd^II^ coordination plane and the eight atoms which belong to both pyridyl ring and imino fragment are coplanar, the larger deviation to the plane is equal to 0.087 (5) and -0.064 (5) Å for O1 and N3 respectively. Only *sp*^3^ carbon C7 and C8 from imino and methyl carbon C9, C10, C11 and C12 deviate significantly from the mean plane.

In the packing, one can notice that intermolecular interactions shorter than the corresponding van-der-Waals radii are only observed between Pd^II^ complexes, no short contact are observed around THF molecule. Centrosymetric contacts take place between imino and pyridyl ring (O1—H4) of one neighbouring complex as well as contacts between chlorine Cl1 and H10B atom of methylene group (Table 1). These contacts yield a two-dimensional network of interacting complexes along the (101) plane (Fig. 2). Another short contact is observed between two adjacent 2-D networks thanks to a van-der-Waals interaction between two C1 atoms of pyridyl rings of two neighbouring molecules with C1—C1 3.393 (16) Å. The shortest distance between two palladium take place between ions from two adjacent planes (Pd1—Pd1 = 3.648 (2) Å), onto a plane, the shorter Pd1—Pd1 distance is 8.7834 (7) Å. THF solvent molecules are lying between two planes. A contact O1s—H1 of 2.7445 Å is observed with pyridyl ring.

### Experimental

Dichlorobis benzonitrile palladium(II) PdCl_2_(PhCN)_2_ and 2-(*ortho*- pyridyl)-4,4,5,5-tetramethyl imidazoline-1-oxyl-3-oxyde (NIT2Py) were synthesized according to literature method (Evans *et al.*, 1968; Ullman & Holm, 1970; Davis *et al.*, 1972). The complex Pd(I*M*_2_py)Cl_2_ was synthesized as follows: The reaction was performed under a dry nitrogen atmosphere using standard schlenk technique. All solvents used were distilled under nitrogen. To a solution of PdCl_2_(PhCN)_2_ (0.1 g;0.26 mmol) in 30 ml of toluene was added with stirring a solution of the radical NIT2Py (0.12 g;0.52 mmol) in 20 ml of toluene. After 2 h of stirring at room temperature, the mixture was filtered and the solvent removed under reduced pressure. Parallelepipedic brown crystals of complex (I) suitable for *x*-ray crystallographic analysis were obtained by slow diffusion of hexane in THF solution of complex (I).

### Refinement

All H atoms were placed in calculated positions and treated as riding model with C—H ranging from 0.93 Å [*U*_iso_(H) = 1.2Ueq(C)] for pyridyl ring to 0.96—0.97 Å with *U*_iso_(H) = 1.5Ueq(C)—1.2Ueq(C)) for methyl and methylene respectively.

### Crystal data

**Table:**

[PdCl_2_(C_12_H_16_N_3_O)]·0.5C_4_H_8_O	*F*_000_ = 1736
*M**_r_* = 431.65	*D*_x_ = 1.707 Mg m^−^^3^
Monoclinic, *C*2/*c*	Mo *K*α radiation λ = 0.71073 Å
Hall symbol: -C 2yc	Cell parameters from 2981 reflections
*a* = 19.1398 (10) Å	θ = 2.6–25.4º
*b* = 15.2061 (12) Å	µ = 1.43 mm^−^^1^
*c* = 13.8291 (10) Å	*T* = 293 (2) K
β = 123.415 (3)º	Thick plate, brown
*V* = 3359.6 (4) Å^3^	0.7 × 0.3 × 0.3 mm
*Z* = 8	

### Data collection

**Table:**

Nonius KappaCCD diffractometer	2049 reflections with *I* > 2σ(*I*)
Radiation source: fine-focus sealed tube	*R*_int_ = 0.059
Monochromator: graphite	θ_max_ = 25.4º
*T* = 293(2) K	θ_min_ = 3.0º
φ and ω scans	*h* = −22→22
Absorption correction: none	*k* = −17→18
5707 measured reflections	*l* = −16→16
3065 independent reflections	

### Refinement

**Table:**

Refinement on *F*^2^	Secondary atom site location: difference Fourier map
Least-squares matrix: full	Hydrogen site location: inferred from neighbouring sites
*R*[*F*^2^ > 2σ(*F*^2^)] = 0.057	H-atom parameters constrained
*wR*(*F*^2^) = 0.152	*w* = 1/[σ^2^(*F*_o_^2^) + (0.0709*P*)^2^ + 4.5052*P*] where *P* = (*F*_o_^2^ + 2*F*_c_^2^)/3
*S* = 1.03	(Δ/σ)_max_ = 0.001
3065 reflections	Δρ_max_ = 0.61 e Å^−^^3^
199 parameters	Δρ_min_ = −0.95 e Å^−^^3^
Primary atom site location: structure-invariant direct methods	Extinction correction: none

### Special details

**Table:**

Experimental. Multiscan absorption correction methods did not yield a better refinement agreement, then no correction was applied.
Geometry. All e.s.d.'s (except the e.s.d. in the dihedral angle between two l.s. planes) are estimated using the full covariance matrix. The cell e.s.d.'s are taken into account individually in the estimation of e.s.d.'s in distances, angles and torsion angles; correlations between e.s.d.'s in cell parameters are only used when they are defined by crystal symmetry. An approximate (isotropic) treatment of cell e.s.d.'s is used for estimating e.s.d.'s involving l.s. planes.
Refinement. Refinement of *F*^2^ against ALL reflections. The weighted *R*-factor *wR* and goodness of fit *S* are based on *F*^2^, conventional *R*-factors *R* are based on *F*, with *F* set to zero for negative *F*^2^. The threshold expression of *F*^2^ > σ(*F*^2^) is used only for calculating *R*-factors(gt) *etc*. and is not relevant to the choice of reflections for refinement. *R*-factors based on *F*^2^ are statistically about twice as large as those based on *F*, and *R*- factors based on ALL data will be even larger.

### Fractional atomic coordinates and isotropic or equivalent isotropic displacement parameters (Å^2^)

**Table:**

	*x*	*y*	*z*	*U*_iso_*/*U*_eq_	
C1	0.4332 (5)	0.2973 (4)	0.6038 (6)	0.0604 (18)	
H1	0.4196	0.3511	0.6214	0.073*	
C2	0.4905 (5)	0.2955 (5)	0.5741 (8)	0.072 (2)	
H2	0.5154	0.3475	0.5723	0.087*	
C3	0.5113 (5)	0.2173 (5)	0.5470 (6)	0.0603 (18)	
H3	0.5497	0.2151	0.5258	0.072*	
C4	0.4733 (5)	0.1419 (4)	0.5520 (6)	0.0558 (18)	
H4	0.4851	0.0877	0.5330	0.067*	
C5	0.4172 (4)	0.1475 (4)	0.5858 (5)	0.0443 (14)	
C6	0.3741 (4)	0.0747 (4)	0.5988 (5)	0.0445 (14)	
C7	0.3264 (5)	−0.0669 (4)	0.6000 (6)	0.0560 (17)	
C8	0.2934 (4)	0.0028 (4)	0.6501 (6)	0.0498 (16)	
C9	0.2604 (6)	−0.0980 (6)	0.4783 (7)	0.081 (2)	
H9A	0.2328	−0.0479	0.4294	0.122*	
H9B	0.2201	−0.1339	0.4806	0.122*	
H9C	0.2867	−0.1317	0.4480	0.122*	
C10	0.3725 (5)	−0.1451 (5)	0.6789 (8)	0.078 (2)	
H10A	0.3899	−0.1843	0.6415	0.117*	
H10B	0.3359	−0.1757	0.6946	0.117*	
H10C	0.4208	−0.1246	0.7503	0.117*	
C11	0.1983 (5)	0.0032 (5)	0.5897 (7)	0.076 (2)	
H11A	0.1824	0.0501	0.6202	0.114*	
H11B	0.1805	−0.0520	0.6030	0.114*	
H11C	0.1723	0.0116	0.5080	0.114*	
C12	0.3364 (5)	−0.0032 (5)	0.7811 (6)	0.067 (2)	
H12A	0.3951	−0.0143	0.8167	0.101*	
H12B	0.3121	−0.0502	0.7994	0.101*	
H12C	0.3292	0.0513	0.8098	0.101*	
N1	0.3961 (3)	0.2250 (3)	0.6084 (5)	0.0470 (12)	
N2	0.3857 (4)	−0.0129 (3)	0.5888 (5)	0.0512 (13)	
N3	0.3211 (3)	0.0884 (3)	0.6270 (5)	0.0509 (13)	
O1	0.4363 (3)	−0.0447 (3)	0.5657 (5)	0.0687 (14)	
Cl1	0.21890 (16)	0.20499 (14)	0.7093 (2)	0.0865 (7)	
Cl2	0.30744 (14)	0.36626 (13)	0.67356 (19)	0.0761 (6)	
Pd1	0.30983 (3)	0.21827 (3)	0.65241 (5)	0.0534 (2)	
O1S	0.5000	0.4980 (7)	0.7500	0.177 (7)	
C2S	0.5251 (8)	0.5487 (7)	0.6926 (10)	0.108 (3)	
H2S1	0.4949	0.5319	0.6116	0.130*	
H2S2	0.5845	0.5408	0.7263	0.130*	
C3S	0.5078 (9)	0.6386 (6)	0.7032 (11)	0.118 (4)	
H3S1	0.4590	0.6595	0.6312	0.142*	
H3S2	0.5552	0.6758	0.7235	0.142*	

### Atomic displacement parameters (Å^2^)

**Table:**

	*U*^11^	*U*^22^	*U*^33^	*U*^12^	*U*^13^	*U*^23^
C1	0.069 (5)	0.050 (4)	0.066 (5)	0.003 (3)	0.040 (4)	−0.004 (3)
C2	0.077 (6)	0.059 (5)	0.091 (6)	−0.023 (4)	0.052 (5)	−0.002 (4)
C3	0.060 (4)	0.068 (5)	0.065 (5)	−0.006 (4)	0.041 (4)	0.000 (4)
C4	0.071 (5)	0.053 (4)	0.064 (5)	0.003 (3)	0.050 (4)	0.003 (3)
C5	0.050 (4)	0.045 (3)	0.039 (3)	−0.002 (3)	0.026 (3)	−0.002 (3)
C6	0.050 (4)	0.046 (3)	0.048 (4)	0.004 (3)	0.034 (3)	0.003 (3)
C7	0.068 (5)	0.046 (3)	0.073 (5)	−0.006 (3)	0.050 (4)	0.001 (3)
C8	0.050 (4)	0.053 (4)	0.054 (4)	0.000 (3)	0.034 (4)	0.008 (3)
C9	0.095 (7)	0.079 (5)	0.083 (6)	−0.018 (5)	0.057 (6)	−0.017 (5)
C10	0.100 (7)	0.060 (5)	0.105 (7)	0.009 (4)	0.075 (6)	0.022 (4)
C11	0.061 (5)	0.085 (6)	0.091 (6)	−0.010 (4)	0.048 (5)	0.000 (5)
C12	0.074 (5)	0.078 (5)	0.060 (5)	−0.003 (4)	0.044 (4)	0.005 (4)
N1	0.051 (3)	0.043 (3)	0.049 (3)	−0.002 (2)	0.029 (3)	0.002 (2)
N2	0.059 (3)	0.043 (3)	0.067 (4)	0.006 (3)	0.044 (3)	0.006 (2)
N3	0.056 (3)	0.052 (3)	0.057 (3)	0.006 (3)	0.039 (3)	0.005 (2)
O1	0.083 (4)	0.052 (3)	0.103 (4)	0.014 (3)	0.072 (4)	0.004 (3)
Cl1	0.0970 (17)	0.0960 (15)	0.1066 (18)	0.0216 (13)	0.0814 (16)	0.0034 (12)
Cl2	0.0873 (15)	0.0578 (10)	0.0865 (15)	0.0200 (10)	0.0499 (13)	−0.0037 (9)
Pd1	0.0586 (4)	0.0552 (3)	0.0549 (4)	0.0116 (3)	0.0366 (3)	−0.0002 (2)
O1S	0.249 (17)	0.068 (6)	0.35 (2)	0.000	0.246 (18)	0.000
C2S	0.116 (9)	0.094 (7)	0.134 (10)	−0.007 (6)	0.081 (8)	−0.014 (7)
C3S	0.178 (12)	0.073 (6)	0.142 (10)	−0.025 (7)	0.113 (10)	−0.009 (6)

### Geometric parameters (Å, °)

**Table:**

C1—N1	1.329 (8)	C10—H10A	0.9600
C1—C2	1.367 (11)	C10—H10B	0.9600
C1—H1	0.9300	C10—H10C	0.9600
C2—C3	1.370 (10)	C11—H11A	0.9600
C2—H2	0.9300	C11—H11B	0.9600
C3—C4	1.381 (9)	C11—H11C	0.9600
C3—H3	0.9300	C12—H12A	0.9600
C4—C5	1.388 (8)	C12—H12B	0.9600
C4—H4	0.9300	C12—H12C	0.9600
C5—N1	1.338 (7)	N1—Pd1	2.052 (5)
C5—C6	1.451 (8)	N2—O1	1.269 (6)
C6—N3	1.289 (7)	N3—Pd1	2.037 (5)
C6—N2	1.370 (8)	Cl1—Pd1	2.280 (2)
C7—N2	1.476 (8)	Cl2—Pd1	2.273 (2)
C7—C9	1.520 (11)	O1S—C2S^i^	1.370 (10)
C7—C10	1.524 (10)	O1S—C2S	1.370 (10)
C7—C8	1.576 (9)	C2S—C3S	1.433 (13)
C8—N3	1.505 (8)	C2S—H2S1	0.9700
C8—C12	1.526 (9)	C2S—H2S2	0.9700
C8—C11	1.529 (10)	C3S—C3S^i^	1.479 (18)
C9—H9A	0.9600	C3S—H3S1	0.9700
C9—H9B	0.9600	C3S—H3S2	0.9700
C9—H9C	0.9600		
			
N1—C1—C2	122.5 (6)	H10B—C10—H10C	109.5
N1—C1—H1	118.8	C8—C11—H11A	109.5
C2—C1—H1	118.8	C8—C11—H11B	109.5
C1—C2—C3	120.1 (7)	H11A—C11—H11B	109.5
C1—C2—H2	120.0	C8—C11—H11C	109.5
C3—C2—H2	120.0	H11A—C11—H11C	109.5
C2—C3—C4	117.9 (6)	H11B—C11—H11C	109.5
C2—C3—H3	121.1	C8—C12—H12A	109.5
C4—C3—H3	121.1	C8—C12—H12B	109.5
C3—C4—C5	119.4 (6)	H12A—C12—H12B	109.5
C3—C4—H4	120.3	C8—C12—H12C	109.5
C5—C4—H4	120.3	H12A—C12—H12C	109.5
N1—C5—C4	121.5 (6)	H12B—C12—H12C	109.5
N1—C5—C6	112.1 (5)	C1—N1—C5	118.6 (6)
C4—C5—C6	126.3 (6)	C1—N1—Pd1	126.5 (4)
N3—C6—N2	112.8 (5)	C5—N1—Pd1	114.9 (4)
N3—C6—C5	120.7 (5)	O1—N2—C6	125.7 (5)
N2—C6—C5	126.4 (5)	O1—N2—C7	123.5 (5)
N2—C7—C9	106.0 (6)	C6—N2—C7	110.6 (5)
N2—C7—C10	109.5 (6)	C6—N3—C8	110.5 (5)
C9—C7—C10	110.5 (7)	C6—N3—Pd1	112.6 (4)
N2—C7—C8	100.9 (5)	C8—N3—Pd1	136.0 (4)
C9—C7—C8	113.9 (6)	N3—Pd1—N1	79.58 (19)
C10—C7—C8	115.1 (6)	N3—Pd1—Cl2	173.28 (15)
N3—C8—C12	106.3 (5)	N1—Pd1—Cl2	93.72 (14)
N3—C8—C11	109.4 (5)	N3—Pd1—Cl1	98.44 (15)
C12—C8—C11	110.5 (5)	N1—Pd1—Cl1	176.70 (15)
N3—C8—C7	102.5 (4)	Cl2—Pd1—Cl1	88.28 (8)
C12—C8—C7	113.4 (6)	C2S^i^—O1S—C2S	111.5 (11)
C11—C8—C7	114.1 (6)	O1S—C2S—C3S	107.6 (9)
C7—C9—H9A	109.5	O1S—C2S—H2S1	110.2
C7—C9—H9B	109.5	C3S—C2S—H2S1	110.2
H9A—C9—H9B	109.5	O1S—C2S—H2S2	110.2
C7—C9—H9C	109.5	C3S—C2S—H2S2	110.2
H9A—C9—H9C	109.5	H2S1—C2S—H2S2	108.5
H9B—C9—H9C	109.5	C2S—C3S—C3S^i^	105.0 (6)
C7—C10—H10A	109.5	C2S—C3S—H3S1	110.7
C7—C10—H10B	109.5	C3S^i^—C3S—H3S1	110.7
H10A—C10—H10B	109.5	C2S—C3S—H3S2	110.7
C7—C10—H10C	109.5	C3S^i^—C3S—H3S2	110.7
H10A—C10—H10C	109.5	H3S1—C3S—H3S2	108.8

Symmetry codes: (i) −*x*+1, *y*, −*z*+3/2.

### Hydrogen-bond geometry (Å, °)

**Table:**

*D*—H···*A*	*D*—H	H···*A*	*D*···*A*	*D*—H···*A*
C4—H4···O1^ii^	0.93	2.61	3.307 (8)	132
C10—H10B···Cl1^iii^	0.96	2.77	3.691 (7)	160

Symmetry codes: (ii) −*x*+1, −*y*, −*z*+1; (iii) −*x*+1/2, *y*−1/2, −*z*+3/2.
